# The association between serum vitamin C levels and respiratory infections in children and adolescents

**DOI:** 10.3389/fnut.2025.1601218

**Published:** 2025-06-05

**Authors:** Ci Li, Zhiwei Zhu, Shicai Jiang, Xiang Feng, Kaijie Gao, Tiewei Li, Liu Yang, Panpan Fang, Junmei Yang

**Affiliations:** Zhengzhou Key Laboratory of Children’s Infection and Immunity, Department of Clinical Laboratory, Children’s Hospital Affiliated to Zhengzhou University, Zhengzhou, China

**Keywords:** vitamin C, respiratory infection, children, adolescents, NHANES5

## Abstract

**Objective:**

Respiratory infections (RIs) are a leading cause of morbidity and mortality, and vitamin C may play a vital role in the risk of RIs. However, high-quality evidence on the association between vitamin C and RIs in the younger population remains limited. This study aimed to investigate the association between serum vitamin C and RI risk in a nationally representative sample of children and adolescents.

**Methods:**

Utilizing data from the National Health and Nutrition Examination Survey (NHANES) 2017–2018, this study included 1,344 children and adolescents aged between 6 and 19 years old. Serum vitamin C levels were obtained from laboratory tests, and RIs were determined based on a self-reported health questionnaire. The association between vitamin C and RIs was tested using multivariable logistic regression models, interaction tests, and smoothing curve fitting.

**Results:**

A total of 238 participants (17.7%) reported a respiratory infection in the past 30 days. Serum vitamin C was significantly and negatively associated with the risk of RIs in all regression models. After adjusting for all potential confounders, an increase of the vitamin C level by 10 units indicated a decrease of the RI risk by 7% (OR = 0.93, 95% confidence interval [CI]: 0.87, 0.99). Such an association remained consistently significant across subgroups with various demographical and health characteristics.

**Conclusion:**

Our study shows a negative association between vitamin C and RIs among children and adolescents, highlighting the protective role of vitamin C against RIs. Our findings suggest that vitamin C supplementation may be potentially used for the prevention and treatment of RIs, which needs to be validated in future well-designed studies.

## 1 Introduction

Respiratory infections (RIs), a broad term including both upper RIs (e.g., common colds, pharyngitis, and sinusitis) and lower RIs (e.g., pneumonia and bronchitis), are a significant public health challenge associated with high morbidity and mortality worldwide (1, 2). Children and adolescents are particularly susceptible to RIs due to their immature immunity systems, which are further aggravated by the increasing environmental pollution in recent years (3). According to the World Health Organization (WHO), pneumonia caused 740,180 deaths among children aged < 5 years in 2019, accounting for 14% of all deaths in that age group (4). Identifying risk factors, especially those modifiable factors that are easy to intervene, is thus crucial for the prevention and control of RIs among children and adolescents.

Abundant evidence has demonstrated that nutrition plays an essential role in RIs among children and adolescents, as poor nutritional status can lead to impaired immunity, thus increasing their susceptibility to RIs (5). Vitamin C is a vital type of micronutrient with anti-inflammatory, antioxidant, and immune-modulating properties (6). It can improve immunity by supporting epithelial barrier integrity, enhancing immune cell activity, and alleviating oxidative stress (7). A multitude of observational studies have indicated a significant association between the level of vitamin C and the risk of RIs, particularly among older adults and people with immune dysfunction (8, 9). In addition, several meta-analyses of randomized controlled trials (RCTs) from global research indicate that vitamin C supplementation can effectively prevent RIs and significantly reduce the duration of RIs (10–12).

However, the relationship between vitamin C and RIs focusing on the younger population who are disproportionally affected by a high disease burden of RIs remains less studied and reported (13). High-quality evidence based on large population studies on the association between vitamin C and RIs in the context of children and adolescents is even scarce. Therefore, this study aimed to evaluate the relationship between serum vitamin C and RIs in a representative sample of children and adolescents from the National Health and Nutrition Examination Survey (NHANES). Our findings would offer comprehensive insights into the impact of nutritional factors on respiratory health in children, providing a basis for developing effective preventive and intervention strategies.

## 2 Methods

### 2.1 Participants

**Figure 1:**
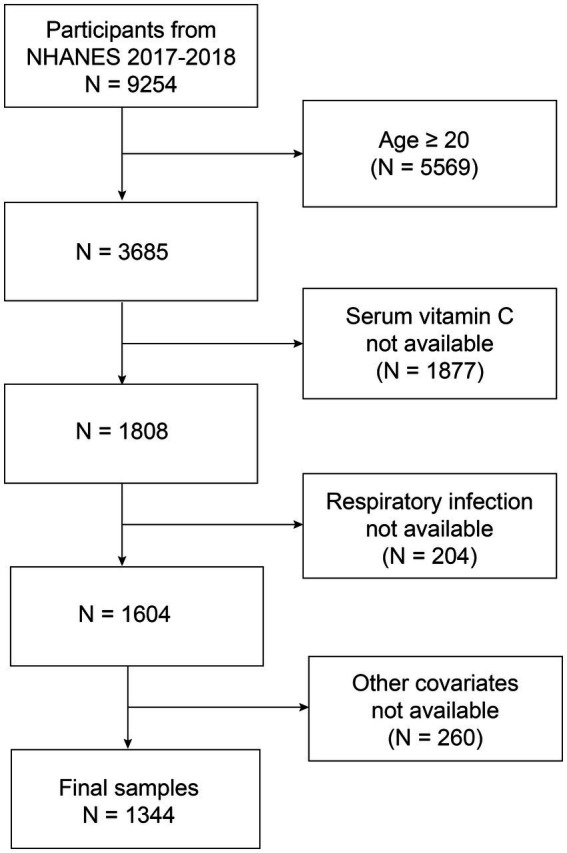
Flow chart of participant selection. NHANES, National Health and Nutrition Examination Survey.

The NHANES is the sole nationwide survey that encompasses health examinations and laboratory testing to investigate the nutrition and health of adults and children in the United States. Ethical approval was obtained from the corresponding Ethics Review Board, and all participants or their legal guardians provided written informed consent before the survey. This study analyzed the 2017–2018 NHANES dataset, including 9,254 participants. We excluded participants who were aged ≥ 20 years old (*n* = 5,569) and those who lacked data on RI status (*n* = 1877), serum vitamin C levels (*n* = 204), and covariates (*n* = 240). Finally, 1,344 participants were included in the analysis (Figure 1).

### 2.2 Exposure variables

The level of serum vitamin C (μmol/L) was assessed by laboratory testing (LBDVICSI) using isocratic ultra-high-performance liquid chromatography coupled with 450 mV electrochemical detection (range: 200 nA). The reported results for serum vitamin C adhered to quality assurance and quality control standards.

### 2.3 Outcome variables

RIs were identified from the following two self-reported questions on the current health status questionnaire (HSQ): (1) HSQ500-Have you caught a cold in the past month? (yes or no), and (2) HSQ520-Have you had an ear infection, pneumonia, or flu in the past month? (yes or no) (14). Participants who answered yes to either question were determined as having RIs.

### 2.4 Covariates

Demographic characteristics and health data were also collected as study covariates. The demographic data set included sex, age, race, and the ratio of family income to poverty (PIR). PIR was calculated by dividing family income by poverty guidelines in the survey year. The cutoffs of 1.3 and 3.5 were used to distinguish between those who were low-income, middle-income, and high-income (15, 16). Health data encompassed body mass index (BMI, kg/m^2^), asthma status, serum cotinine levels (ng/mL), total energy intake (kcal), and vitamin C intake (mg). BMI was derived from examination data and calculated as weight in kilograms divided by height in meters squared (kg/m^2^). The cutoffs of 25 and 30 were used to distinguish between those who were normal, overweight, and obese (17). Asthma status was determined from questionnaire data, specifically MCQ010-whether the participant had ever been told they had asthma (yes or no). Serum cotinine levels (ng/mL) were obtained from laboratory data to assess exposure to tobacco smoke, with a cutoff of 0.05 ng/mL distinguishing between those with and without tobacco smoke exposure (18, 19). Total energy intake (kcal) was the mean value of the total energy intake on day 1 and day 2 from the questionnaire data. The vitamin C intake (mg) was the mean value of vitamin C intake on day 1 and day 2 from the questionnaire data.

### 2.5 Statistical analysis

The association between the level of serum vitamin C and RI risk was assessed using multivariable logistic regression analysis and trend tests with various models. Model 1 was the crude model without adjusting for any covariates. Model 2 was the partially adjusted model controlling for sex, age, and race. Model 3 was the fully adjusted model that further controlled for PIR, BMI, asthma, tobacco smoke exposure, total energy intake, and vitamin C intake, which was visually presented using the smoothing curve fitting. To evaluate the stability of the association between serum vitamin C and RI risk, we conducted multiple exploratory subgroup analyses in various subgroups with different characteristics. Statistical analyses were conducted using EmpowerStats (version 4.2) and R software (version 4.3). Statistical significance was indicated by *p* < 0.05.

## 3 Results

### 3.1 Sample characteristics

The study included 1,344 participants, and their mean age was 12.62 ± 3.90 years. Among them, 670 were male, accounting for 49.85%. Furthermore, 238 participants (17.7%) reported a history of RIs within the past 30 days. Participants were categorized into four groups based on quartiles of serum vitamin C levels: Q1 < 43.1 μmol/L (*n* = 336), Q2 = 43.2–62.5 μmol/L (*n* = 330), Q3 = 62.6–78.9 μmol/L (*n* = 337), and Q4 > 79.0 μmol/L (*n* = 341). Table 1 shows the comparison of sample characteristics by serum vitamin C quartiles, which showed significant differences in age, sex, BMI, tobacco smoke exposure, and vitamin C intake among the four groups. Compared to the lower serum vitamin C quartile groups, the higher quartile groups tended to be younger (*p* < 0.001) and had more females (*p* = 0.045), lower BMI (*p* < 0.001), less tobacco smoke exposure (*p* < 0.001), and higher Vitamin C intake (*p* < 0.001).

**Table 1:** Basic characteristics of participants by serum vitamin C quartiles among U. S. children and adolescents.

Characteristics	Serum vitamin C quartiles				*p*-value
	Q1 (*N* = 336)	Q2 (*N* = 330)	Q3 (*N* = 337)	Q4 (*N* = 341)	
Age (years) (mean ± SD)	14.44 ± 3.23	13.27 ± 3.74	12.17 ± 3.90	10.64 ± 3.67	<0.001
Sex, (n, %)					0.045
Male	186 (55.36%)	170 (51.52%)	153 (45.40%)	161 (47.21%)	
Female	150 (44.64%)	160 (48.48%)	184 (54.60%)	180 (52.79%)	
Race/ethnicity, (%)					0.128
Mexican American	17.56	18.48	18.99	18.18	
Other Hispanic	5.65	6.97	9.20	6.45	
Non-Hispanic White	34.52	27.88	28.49	31.96	
Non-Hispanic Black	18.15	25.76	22.55	26.69	
Other Race	24.11	20.91	20.77	16.72	
Family PIR (mean ± SD)	2.04 ± 1.54	2.12 ± 1.53	2.12 ± 1.48	2.13 ± 1.53	0.587
BMI (kg/m^2^)	24.88 ± 7.52	23.25 ± 6.73	21.84 ± 5.96	19.65 ± 4.80	<0.001
Asthma, (%)					0.586
Yes	16.37	19.70	19.88	17.60	
No	83.63	80.30	80.12	82.40	
Tobacco smoke exposure, (%)					<0.001
Yes	51.95	41.80	38.14	37.28	
No	48.05	58.20	61.86	62.72	
Total energy intake, (mean ± SD)	1988.57 ± 1007.54	2054.08 ± 931.94	2026.66 ± 866.54	1946.23 ± 753.02	0.483
Vitamin C intake, (mean ± SD)	43.53 ± 44.31	64.42 ± 58.06	74.79 ± 51.45	87.22 ± 60.20	<0.001

Data are presented as the means ± standard deviations for continuous variables. PIR, ratio of income to poverty, BMI, body mass index.

### 3.2 Associations between serum vitamin C and RI risk

**Figure 2:**
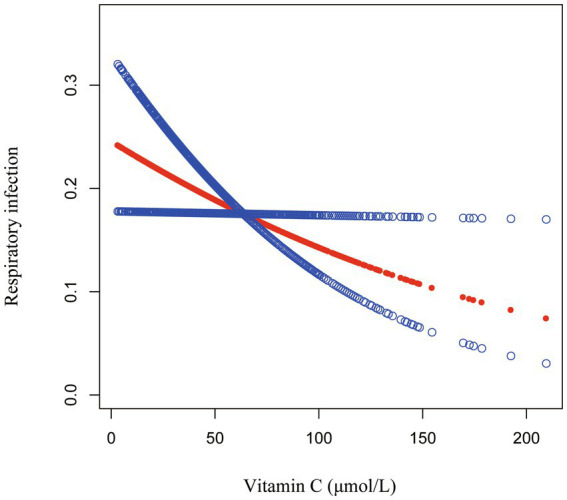
Associations between serum vitamin C and respiratory infection. The solid red line represents the smooth curve fit between the variables, while the blue bands denote the 95% confidence intervals of the fitted values. Age, sex, race, the ratio of family income to poverty, body mass index, asthma, tobacco smoke exposure, total energy intake, and vitamin C intake were adjusted.

Table 2 shows the associations between serum vitamin C and RI risk in various models. A significant negative association between the continuous serum vitamin C level and RI risk was only observed in model 3, where a 10-unit increase in serum vitamin C was associated with a 7% decreased risk of RIs (OR = 0.93, 95% CI: 0.87, 0.99). Additionally, compared to the Q1 group, only the Q4 group showed a significantly lower risk of RIs in Model 2 and Model 3. In Model 3, the Q4 group had a 50% lower risk of RIs than the Q1 group (OR = 0.50, 95% CI: 0.30, 0.83). This result was further visually presented using the smoothing curve fitting (Figure 2). Our results indicated that the risk of RIs decreased as serum vitamin C levels increased (P for trend < 0.05).

**Table 2:** Associations between serum vitamin C levels and respiratory infection in different models.

Serum vitamin C	Respiratory infection OR (95% CI)		
	Crude model (Model 1)	Partially adjusted model (Model 2)	Fully adjusted model (Model 3)
Continuous variables (μmol/L)	0.97 (0.92, 1.02)	0.95 (0.90, 1.00)	0.93 (0.87, 0.99)
Categorical variables			
Quartile 1	1 (ref)	1 (ref)	1 (ref)
Quartile 2	1.13 (0.77, 1.65)	1.08 (0.73, 1.60)	0.99 (0.65, 1.52)
Quartile 3	0.98 (0.66, 1.44)	0.88 (0.58, 1.32)	0.75 (0.48, 1.18)
Quartile 4	0.72 (0.48, 1.09)	0.61 (0.39, 0.95)	0.50 (0.30, 0.83)
P for trend	0.099	0.019	0.003

Model 1: no covariates were adjusted. Model 2: age, sex, and race were adjusted. Model 3: age, sex, race, PIR, BMI, asthma, tobacco smoke exposure, total energy intake, and vitamin C intake were adjusted. Abbreviations: PIR, ratio of income to poverty, BMI, body mass index.

### 3.3 Subgroup analyses

After adjusting for all covariates, subgroup analysis was conducted to evaluate the stability of the association between the serum vitamin C level and RI risk across different subgroups with various characteristics. As shown in Table 3, the relationship between serum vitamin C and RIs remained stable across various subgroups, including sex, age, PIR, BMI, asthma, and tobacco smoke exposure (P for interaction > 0.05). Additionally, a significant negative association between serum vitamin C and RI risk was only observed in males (OR = 0.90, 95% CI: 0.82, 0.99) and participants with PIR ≤ 1.3 (OR = 0.89, 95% CI: 0.80, 0.98).

**Table 3:** Subgroup analysis of the association between serum vitamin C (μmol/L) and respiratory infection.

Subgroup	Respiratory infection [OR (95%CI)]	P for interaction
Sex		0.291
Male	0.90 (0.82, 0.99)	
Female	0.96 (0.88, 1.05)	
Age		0.927
< 12 years	0.93 (0.85, 1.03)	
≥ 12 years	0.94 (0.86,1.02)	
Family PIR		0.311
≤ 1.3	0.89 (0.80, 0.98)	
1.3–3.5	0.99 (0.89, 1.10)	
> 3.5	0.95 (0.83, 1.09)	
BMI		0.312
< 24.9 kg/m^2^	0.95 (0.88, 1.02)	
25–29.9 kg/m^2^	0.79 (0.62, 1.01)	
≥ 30 kg/m^2^	0.88 (0.72, 1.06)	
Asthma		0.217
Yes	0.85 (0.73, 1.00)	
No	0.95 (0.89, 1.02)	
Tobacco smoke exposure		0.585
Yes	0.92 (0.84, 1.01)	
No	0.95 (0.87, 1.04)	

Age, sex, race, the family PIR, BMI, asthma, tobacco smoke exposure, total energy intake, and vitamin C intake were adjusted. Abbreviations: PIR, ratio of income to poverty, BMI, body mass index.

## 4 Discussion

This was the first population-based study examining the association between serum vitamin C levels and RIs in US children. After adjusting for demographic and health characteristics, we found that a higher level of serum vitamin C was associated with a lower risk of RIs. Subgroup analyses further substantiated this association, demonstrating its robustness and resistance to influence by confounding variables such as sex, age, family PIR, BMI, asthma, or tobacco smoke exposure. Our findings highlight the protective role of vitamin C against RIs, which may provide useful guidance for future prevention and treatment of RIs among children.

The finding that higher serum vitamin C was associated with a RI risk in children and adolescents was consistent with previous studies in other countries. A survey conducted in the United Kingdom involving 19,357 adults found an inverse relationship between vitamin C and the risk of RIs (20). A study conducted in Italy, which involved 60 children, found that supplementing vitamin C in children significantly decreased the incidence of recurrent RIs (21). A similar finding was reported in a study conducted among 69 preschool children in Slovakia, which demonstrated that combining vitamin C and probiotics significantly decreased the incidence of upper RIs and the use of antibiotics and cough medications (22). Our study added further evidence on the beneficial effects of vitamin C in RI prevention and control, which could inform future research and clinical applications.

The underlying mechanisms of the beneficial role of vitamin C on RIs primarily involve its antioxidant, immune-modulatory, and anti-inflammatory properties. Research indicates that vitamin C protects against RIs through several mechanisms. First, vitamin C exerts antioxidant functions by neutralizing free radicals, mitigating oxidative stress, and protecting the integrity of respiratory epithelial cells (7). Second, vitamin C strengthens immunity by enhancing the chemotaxis and phagocytic activity of neutrophils, which is essential for pathogen clearance (23). Third, vitamin C exerts anti-inflammatory functions by modulating cytokine production and inhibiting pro-inflammatory cytokines (e.g., TNF-αand IL-6), thereby reducing inflammatory responses (24). Fourth, our previous research has demonstrated a negative correlation between serum vitamin C and C-reactive protein (CRP), an important inflammatory marker, in children (13). Vitamin C can reduce pulmonary inflammation, improve respiratory function, and shorten the duration of the RIs through its anti-inflammatory functions (25).

On the other hand, vitamin C deficiency can increase the risk of RIs. Previous studies have observed lower levels of vitamin C in hospitalized patients with RIs, probably due to increased metabolic demands (26). In addition, patients with lower vitamin C levels exhibit more severe symptoms and longer recovery duration during infections (27). In critically ill patients, intravenous supplementation of vitamin C can reduce the length of hospitalization and improve survival (28). Consequently, ensuring adequate vitamin C supplementation holds potential clinical significance in the management of RIs, particularly among vulnerable populations, such as older adults and immunocompromised individuals (29). In our study, maintaining higher serum vitamin C concentrations was crucial in preventing RIs in children and adolescents.

The present study boasts several strengths. First, participants were recruited from the 2017–2018 NHANES, ensuring the sample representativeness. Second, the analysis was adjusted for multiple potential confounders, enhancing the reliability of the findings. Third, the results were tested among various subgroups, confirming the stability of the association. However, the study has several limitations. First, the sample was recruited from a US population and may not represent children in other countries. Future studies in other nations are needed to validate our findings in other populations. Second, the cross-sectional study design cannot establish causal references, indicating the need for future longitudinal study designs. Third, although we adjusted for multiple confounders, there may be other unmeasured potential confounding factors that affect the results. For instance, we did not account for other drugs that may influence vitamin C absorption, depletion, or demand, which may make it difficult to determine the true effect of vitamin C in our study. Future research should address potential drug interactions to provide more accurate and reliable conclusions about the role of vitamin C in RIs. Fourth, RIs were determined based on two self-reported questions, which may introduce potential information and recall bias. Future studies should consider using more robust assessment approaches, such as medical records, physical examinations, and laboratory tests to get a more accurate evaluation. Fifth, while our study showed that lower vitamin C levels were associated with a higher risk of RIs, it should be noted that RIs may also lead to lower vitamin C levels due to the increased demands of the immune system during the infection response. Future research should consider exploring the bidirectional relationship between vitamin C levels and RIs and their underlying mechanism, offering a more nuanced understanding of their interactions. Finally, we did not account for other health conditions that may impair vitamin C absorption or metabolism. Conditions like irritable bowel disease (IBD), celiac disease, and chronic infections can all contribute to vitamin C deficiency through various ways, including reduced nutrient intake, increased nutrient losses, and impaired absorption. Future research should consider and control for these factors to improve the accuracy and relevance of findings.

## 5 Conclusion

This study demonstrates a negative association between serum vitamin C and RI risk in a nationally representative sample of children and adolescents. These findings highlight the protective role of vitamin C against RIs and underscore the significance of maintaining optimal vitamin C levels. Our findings suggest that vitamin C supplementation may be potentially used for the prevention and treatment of RIs among children and adolescents, which needs to be further validated in future well-designed studies.

## Data Availability

Publicly available datasets were analyzed in this study. This data can be found at: www.cdc.gov/nchs/nhanes/.
